# Preparation of bioactive glass nanoparticles with highly and evenly doped calcium ions by reactive flash nanoprecipitation

**DOI:** 10.1007/s10856-021-06521-x

**Published:** 2021-04-23

**Authors:** Lijun Ji, Tong Xu, Jun Gu, Qingren Liu, Shu Zhou, Guojun Shi, Zhengxi Zhu

**Affiliations:** 1 School of Chemistry and Chemical Engineering, Yangzhou University, Yangzhou, 225002 China; 2 Department of Orthopaedics, Xishan People’s Hospital of Wuxi, Wuxi, 214000 China; 3 Department of Anesthesiology, Xishan People’s Hospital of Wuxi, Wuxi, 214000 China

## Abstract

Nanoscale bioactive glass particles have greater bioactivity than microscale bioactive glass particles, due to their high-specific surface area and fast ion release rate in body fluid. However, preparation of bioactive glass nanoparticles (BGNPs) is difficult since calcium is not easy to be highly doped into the forming silica atom network, leading to an uneven distribution and a low content of calcium. In addition, BGNPs are usually prepared in a dilute solution to avoid agglomeration of the nanoparticles, which decreases the production efficiency and increases the cost. In this work, BGNPs are prepared by a method of the reactive flash nanoprecipitation (RFNP) as well as a traditional sol–gel method. The results indicate that the BGNPs by the RFNP present a smaller size, narrower size distribution, more uniform composition, and better bioactivity than those by the traditional sol–gel method. The obtained BGNPs have uniform compositions close to the feed values. The high and even doping of calcium in the BGNPs is achieved. This successful doping of calcium into nanoparticles by the RFNP demonstrates a promising way to effectively generate high-quality BGNPs for bone repairs.

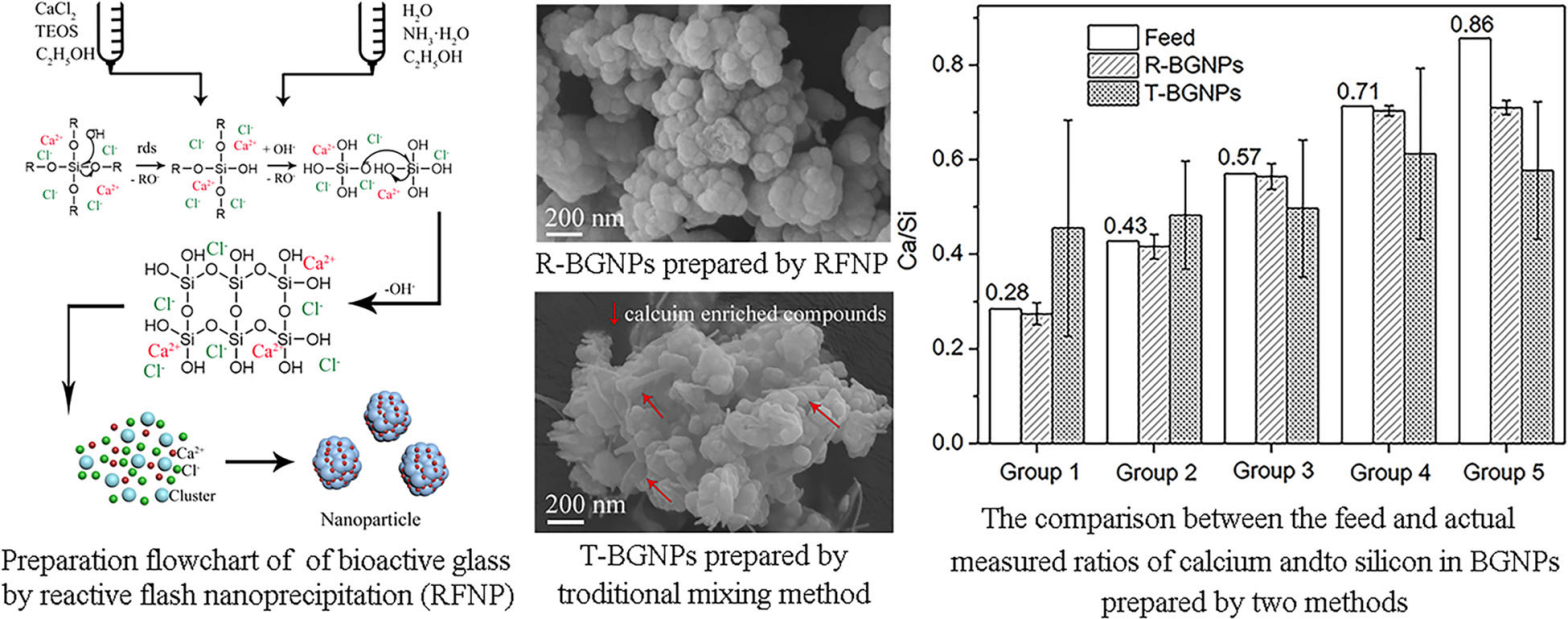

## Introduction

Bone repair materials are in high demand due to the aging population and an enhanced incidence of trauma diseases [1]. An autologous bone, allograft and artificial bone can be used for repairing bone defects in clinic. However, a transplantation of autologous bones is limited by their sources and possible bone infection. An allograft may result in immune rejection and virus transmission, as well as raising questions of medical ethics. Therefore, developing artificial materials for repairing bones has become an important research area [2, 3]. Bone is an organic–inorganic nanocomposite with a hierarchical structure, mainly composed of collagen and hydroxyapatite nanocrystals. As a repairing material for bones, not only the components but also the nano-structure has to be mimicked to meet bone repairs [4].

Bioactive glass (BG) particles could be a potential material to mimic the hydroxyapatite nanocrystals in bones due to their excellent osteogenic properties and self-degradation properties [5–7]. They have been incorporated with biopolymers extensively to prepare three dimensional macroporous scaffolds [8–10] and drug delivery systems [11, 12]. It has been shown that the particle size can affect the BG properties significantly. Nano-sized BG particles usually show better bioactive properties than micro-sized ones, because their high-specific surface area is beneficial in ensuring sufficient release of ions [6, 7, 13]. The methods used for preparing nano-sized BG particles include the sol–gel and coprecipitation methods [14, 15], the surfactant assisted sol–gel method [16–18], the microemulsion method [19–21], the modified Stöber method [22], and the template synthesis method [23]. However, preparation of BG nanoparticles (BGNPs) at a high concentration is quite difficult because the primary particles formed easily agglomerate to form large particles, making the BG particles quite large in size and with a broad size distribution. In order to avoid such agglomeration, nano-sized BG particles are usually prepared in a dilute solution, which reduces production efficiency and raises production cost. Moreover, it is hard to dope a high content of ions evenly into an atom network of silica, since the water-soluble ions are not easily to be trapped fast and effectively by the forming silica network, and rather rapidly phase separate from the forming silica particles. Either the particle size or the ion doping issue has greatly limited the application of BGNPs in the healthcare industry.

The flash nanoprecipitation (FNP) is a technique capable of producing nanoparticles continuously and rapidly. The process is simple and able to be scaled up easily. The nanoparticles are precipitated out inside a tiny and confined chamber by turbulence mixing and solvent shifting. The mixer used for the FNP has been developed for three generations. The first generation of the FNP mixer, a confined impinging jet (CIJ) mixer, was designed by Johnson and Prud’homme and had two inlets [24]. Therein, two fluids were simultaneously injected into the mixer by a high-pressure injection pump at an approximately equal momentum. The second generation, a multi-inlet vortex mixer (MIVM) [25, 26], allowed unequal momentums of the injected streams. However, due to complicated internal flow channels, the pressure drop of the MIVM was very high, and not good for further enhancing flow rate and scaling-up. The third-generation of the mixer, a CIJ with dilution (CIJ-D) mixer [27], was designed by Han et al. and allowed unequal momentums of two injected streams. The flow channels were well optimized, and the pressure drop was quite low [28]. The two streams can even be injected into the mixer by pushing the syringes with fingers. Zhu et al. combined the FNP technique with an in situ chemical reaction and proposed the reactive FNP (RFNP) [29], and the competitions between turbulence mixing and chemical reactions were investigated [30]. Zhu et al. further employed the CIJ-D mixer into the RFNP and realized a fast generation and separation of nanoparticles, which was promising for large-scale industrial production [31]. Since the fluids were mixed evenly in the chamber within tens of microseconds, the succeeding chemical reaction and phase separation (i.e., solid nanoparticles form in an aqueous medium) can start evenly and instantly. The generated particles would thus have a small size and narrow size distribution.

**Fig. 1:**
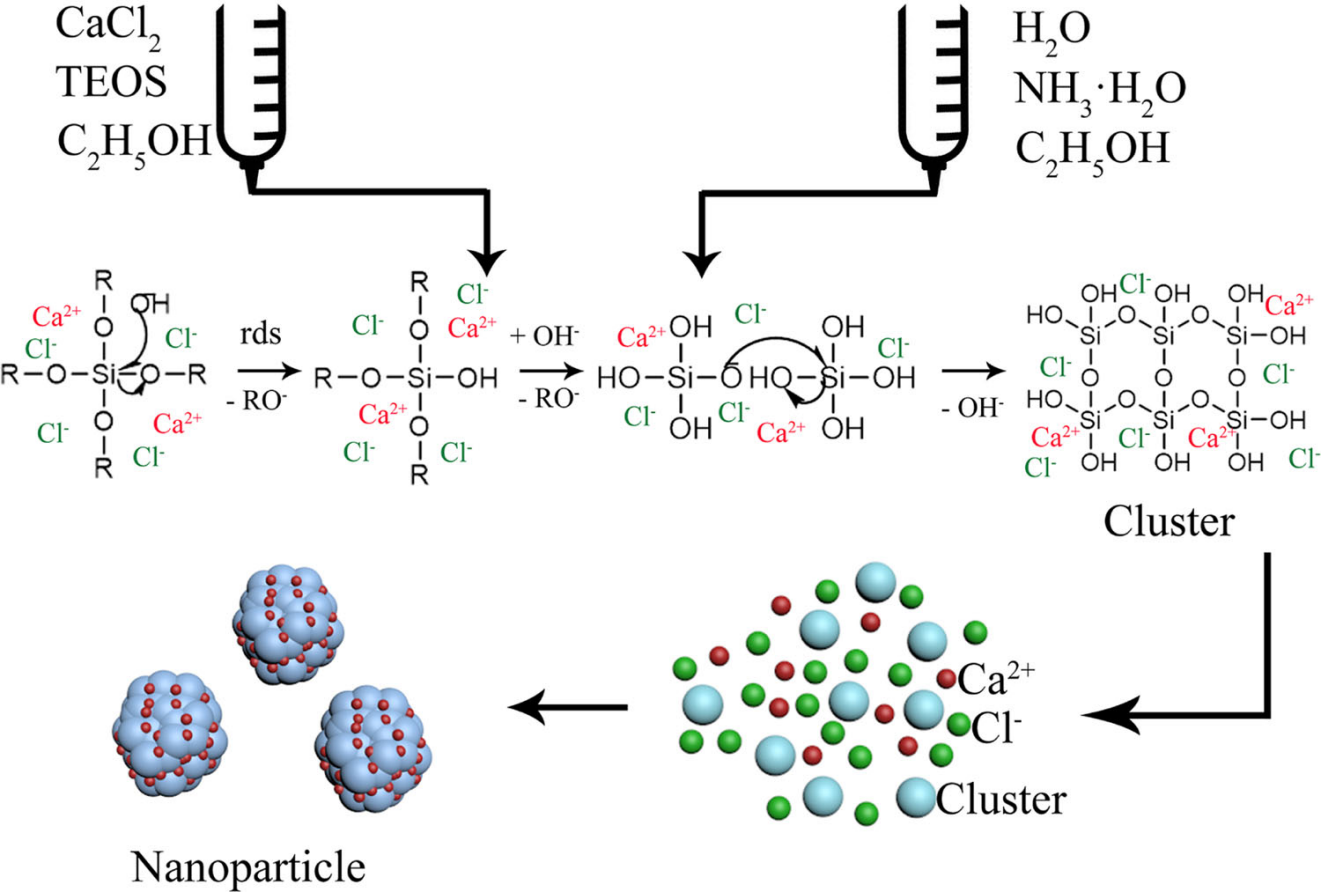
Scheme of preparation of bioactive glass nanoparticles via the RFNP

Inspired by the above work, as shown in Fig. 1 we herein propose to prepare BGNPs via the RFNP with a CIJ-D mixer by mixing ammonia in a water/ethanol mixture with tetraethyl orthosilicate (TEOS) and calcium chloride (CaCl_2_) in ethanol. In the mixer chamber, the two solutions mix instantly and evenly. TEOS hydrolyzes and converts into SiO_2_, which would precipitate out to form nanoparticles. Since oxygen atoms could attract calcium atoms in some degree, Ca can coprecipitate with SiO_2_. Seldom calcium compounds including calcium nitrate and calcium chloride can be used as a calcium source for a BG synthesis [32]. Calcium nitrate is toxic to human, and a BG sample prepared with it must be heated over 600 °C to decompose it. Such high temperature could cause significant agglomeration of the BGNPs. Consequently CaCl_2_ was chosen due to its nontoxicity, and for the fact that the last step of high temperature processing was no longer necessary. However, like other calcium salts, Ca^2+^ is easy to be phased out of the BG during a particle preparation [33], which led to an uneven distribution of calcium inside and outside the BG particles and a large deviation of the local composition from the feed ratio, especially for doping with high calcium levels. Herein, we anticipate that the instant mixing and sufficiently fast coprecipitation of calcium with silica via the RFNP can solidify their blend state and repress the separation of Ca^2+^ from the BGNPs, so as to obtain BGNPs with an uniform composition close to the feed ratio, which is important for producing high-quality BGNPs. Moreover, it is desirable to use high concentrations of the reactants, so that the rates of reaction and particle formation are high. A high reactant concentration is also beneficial for improving the production efficiency and lowering the cost of BGNPs, which is a key in an industrial production process. The size, size distribution, composition, and bioactivity of the BGNPs prepared by the RFNP (notated as R-BGNPs) would then be compared with those by the traditional sol–gel method (notated as T-BGNPs) to confirm the quality of the generated BGNPs with a calcium doping.

## Materials and methods

### Preparation of bioactive glass nanoparticles

The BGNPs in the Ca/Si feed molar ratios of 0.28, 0.36, 0.43, 0.57, 0.71, and 0.86 were prepared by either the RFNP or the traditional sol–gel method. All reagents were purchased from the Sinopharm Chemical Reagent and used as received. For a typical formula with a Ca/Si feed molar ratio of 0.43, ammonia, anhydrous ethanol, and deionized water were added into a beaker in a mass ratio of 1:2:2 and stirred. This solution was notated as solution A. In total, 6.0 g of ethanol, 1.458 g of TEOS, and 0.222 g of CaCl_2_ were added into another beaker and stirred until TEOS and CaCl_2_ were dissolved completely in ethanol. The solution was notated as solution B. In total, 5.0 ml of each solution was then loaded into an individual gas-tight plastic syringe connected with an individual inlet of the CIJ-D mixer. The mixer used in this study was the same as the one used in our previous RFNP work [31]. The two syringes were propelled at the same speed and solutions were injected into the mixer at the same flow rate of about 1.0 ml/s. The mixing Reynolds number was ~9000, which was much higher than the critical value of ~950, ensuring a sufficient turbulence mixing for achieving an asymptotic and minimized particle size [34]. Two solutions reacted inside the chamber under turbulent mixing, and the BGNPs were formed. The suspension of BGNPs flowed out from the outlet tubing of the mixer and was collected with a beaker. In addition, the traditional sol–gel method was carried out by mixing the same two solutions as those used in the RFNP in a beaker. Each of 5.0 ml of solution was loaded in a syringe and injected into a beaker at the same time within 5 s and then stirred for 1 h. For both methods, the suspension obtained was centrifuged and washed with water three times. The precipitates were freeze dried, and white powders of BGNPs were then obtained.

### Material characterization

The surface morphology of the prepared BGNP powder was observed by a field emission scanning electron microscope s4800-ii (SEM, Philips), and energy dispersive spectroscopy (EDS) data were obtained at 20 kV to determine the composition of the samples. Five different regions of the sample surface were selected for EDS analysis, and a mean value and a standard deviation were calculated. The size and size distribution of a BG suspension were measured by dynamic light scattering (DLS) at a wavelength of 632.8 nm, a scattering angle of 90° (Nano-ZS90, Malvern Instruments), and room temperature. Each sample was measured three times, and an intensity averaged particle size and polydispersity index (PDI) showing a width of the size distribution were obtained.

An in vitro biological activity of the BG particles was determined by immersing a BG powder sample in a simulated body fluid (SBF), a water solution of various inorganic ions in a concentration close to that in human blood plasma, for evaluating the formation of hydroxyapatite on the BGNPs [35]. The SBF is buffered by tris(hydroxymethyl)aminomethane and hydrochloric acid. The BGNP powder was soaked in an SBF solution in 1.0 mg/ml, and then the suspension was placed on a shaking bed at 37 °C and 170 rpm. A sample was removed from the shaking bed after 1, 3, 7, and 14 days, respectively. BGNPs were separated by centrifugation from the SBF solution, vacuum dried overnight at 60 °C after washing by water, and further characterized by SEM, Fourier transform infrared spectra (FTIR), X-ray diffraction (XRD), and inductively coupled plasma (ICP). The crystal structure of the sample was determined by XRD with a D8 Advance (Bruker) X-ray diffractometer. In order to determine the biodegradation of the BGNPs, the ionic components in an SBF solution were measured with ICP. FTIR were obtained by an FTIR spectrometer (Bruker Tensor 27).

## Results and discussion

**Fig. 2:**
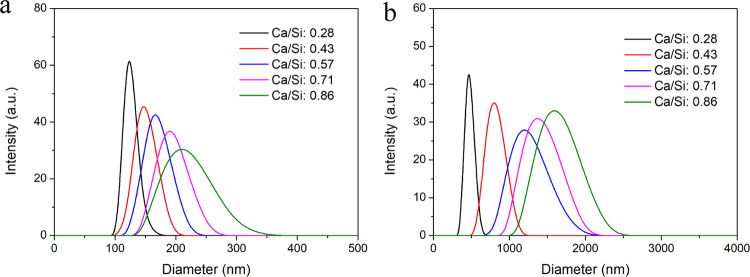
The particle size distribution (measured by DLS) of the BGNP suspension prepared by (**a**) the RFNP and (**b**) the traditional mixing with various feed molar ratios of Ca/Si

The DLS measurements indicate that the particle sizes of the R-BGNPs prepared according to different formulations distributed in the range of 100–350 nm (Fig. 2a), while those of the T-BGNPs had a much wider distribution, from 300 to 2000 nm (Fig. 2b). The average particle sizes (PDI in brackets) of the R-BGNPs were 132 (0.25), 151 (0.25), 173 (0.27), 212 (0.26), and 253 nm (0.28), respectively, which were much smaller than the corresponding ones of the T-BGNPs, i.e., 481 (0.31), 773 (0.32), 1491 (0.35), 1771 (0.34), and 1939 nm (0.29), respectively. This contrast is consistent with our expectations that the instant mixing via the RFNP would be helpful for making the succeeding hydrolyzation and particle formation evenly occur. The particle size in the RFNP shall be smaller and narrower distributed than the one in a traditional mixing with a relatively slow and uneven effect.

**Fig. 3:**
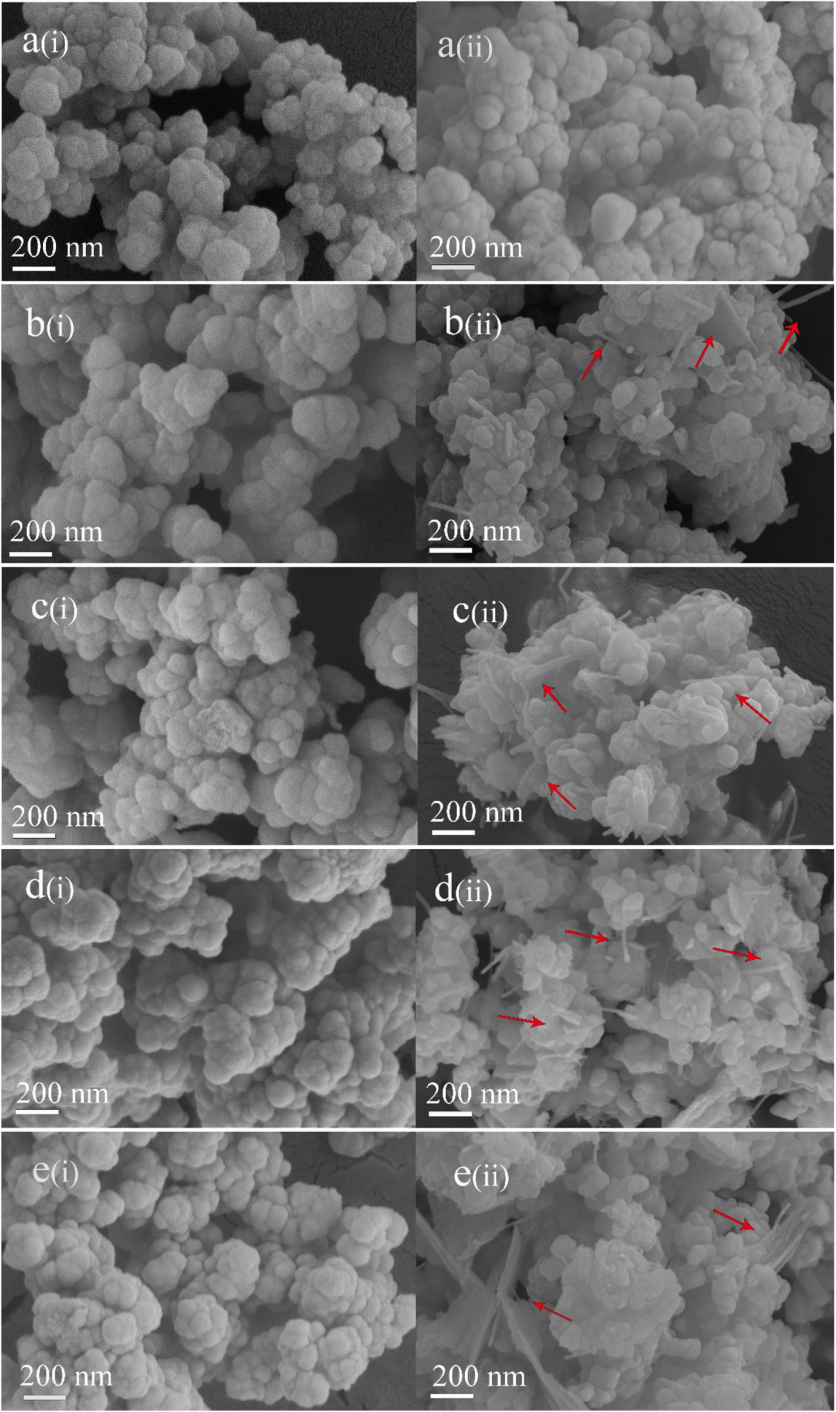
The SEM images of BGNPs with feed molar ratios of Ca/Si in 0.28, 0.36, 0.43, 0.57, 0.71, and 0.86 prepared by either the RFNP or the traditional mixing: **a**(i)–**e**(i) R-BGNPs; **a**(ii)–**e**(ii) T-BGNPs

**Fig. 4:**
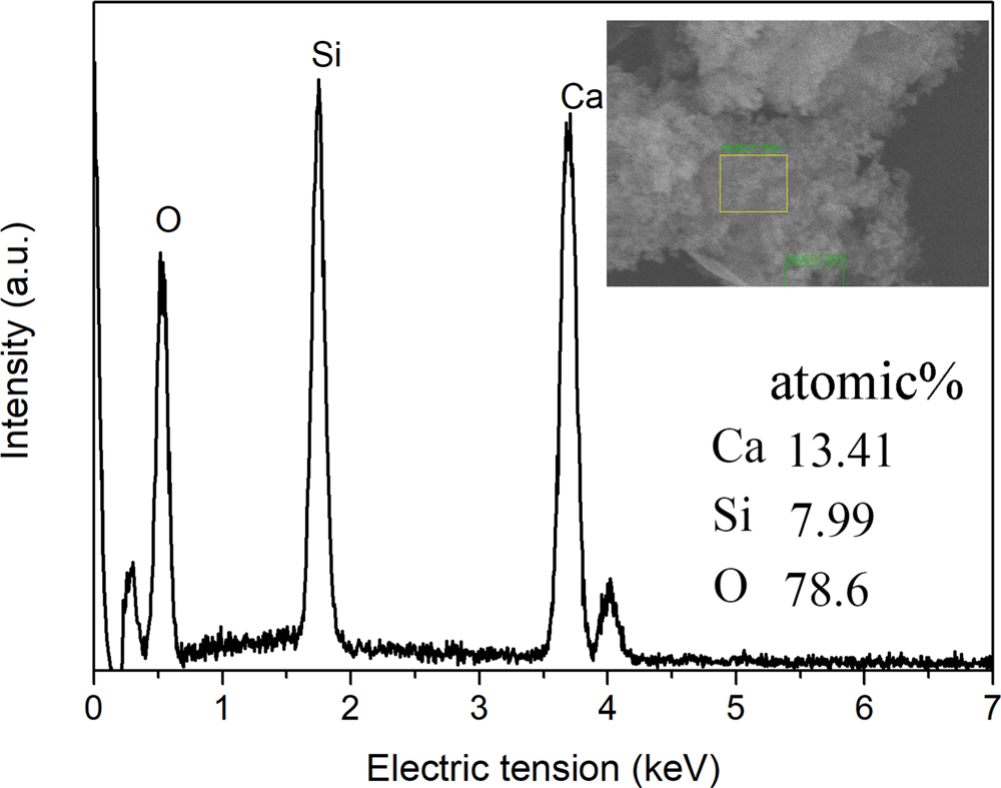
The EDS spectrum of the rod-shaped BGNPs prepared by the tradition mixing with a feed molar ratio of Ca/Si in 0.43

Figure 3 shows the SEM images of the R-BGNPs and T-BGNPs with various feed ratios of Ca/Si. The R-BGNPs in Fig. 3a(i)–e(i) present aggregates with the primary particles in a nearly spherical shape smaller than ~100 nm in diameter. In contrast, the T-BGNPs in Fig. 3a(ii)–e(ii) present aggregates of not only spheres but also sheets and rods (see arrows in the images). As the feed ratio of Ca/Si increases, more sheet- or rod-shaped particles appear, implying that these non-spherical particles might be calcium enriched compounds. An EDS measurement focusing on the sheets and rods of the T-BGNPs confirms that the measured Ca/Si ratio of 1.67 therein is much higher than the feed one of 0.43 (Fig. 4). Calcium is not as evenly distributed in the T-BGNPs as in the R-BGNPs.

**Fig. 5:**
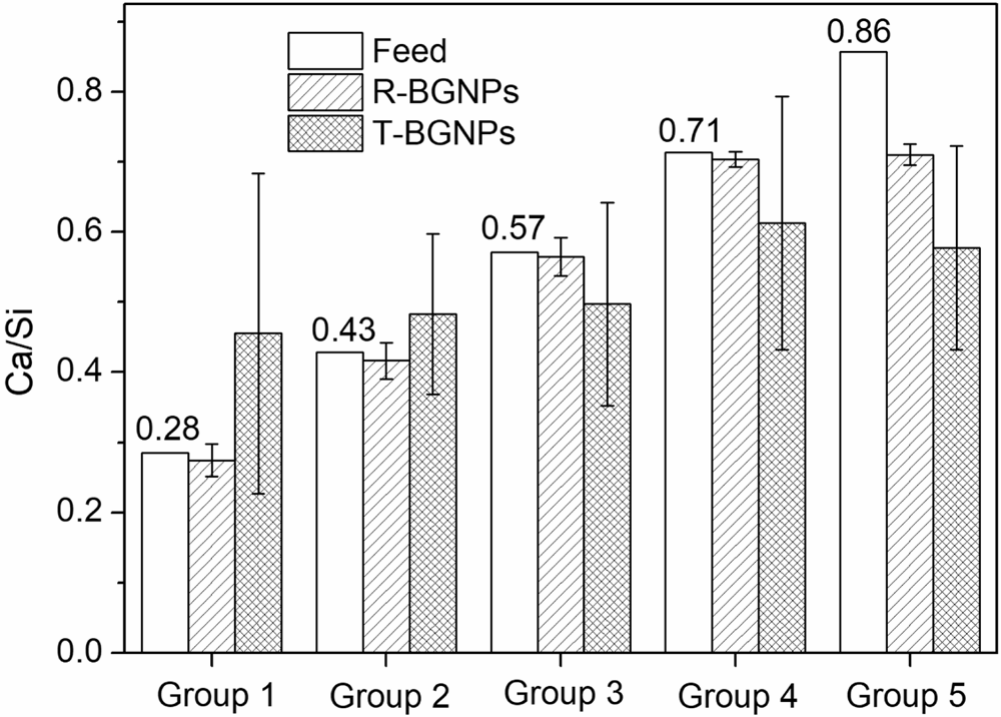
The comparison between the feed and measured molar ratios of Ca/Si in the R-BGNPs or T-BGNPs

For all of the T-BGNP powders, a significant standard deviation of the EDS measurements occurs (Fig. 5), indicating an uneven distribution of Ca^2+^ in the specimens. By contrast, for the R-BGNP powder, the measured average value of Ca/Si has a small deviation as well as being close to the feed ratio, except for the sample with a very high feed Ca/Si of 0.86. This small differentiation and small deviation indicate that Ca^2+^ is evenly distributed in the R-BGNP specimens. For a high Ca/Si ratio, more of the Ca^2+^ might not be able to be trapped in the SiO_2_ matrix, so that a small portion of Ca^2+^ would stay in water and be washed away during the separation. In spite of this, the R-BGNPs still have a higher average of the measured Ca/Si than the T-BGNPs. The Ca^2+^ distribution in the R-BGNPs is much more even than the distribution in the T-BGNPs. The fast mixing and succeeding chemical reaction via the RFNP speed up the formation of the SiO_2_ particles, facilitating more Ca^2+^ being wrapped into the forming SiO_2_ matrix.

**Fig. 6:**
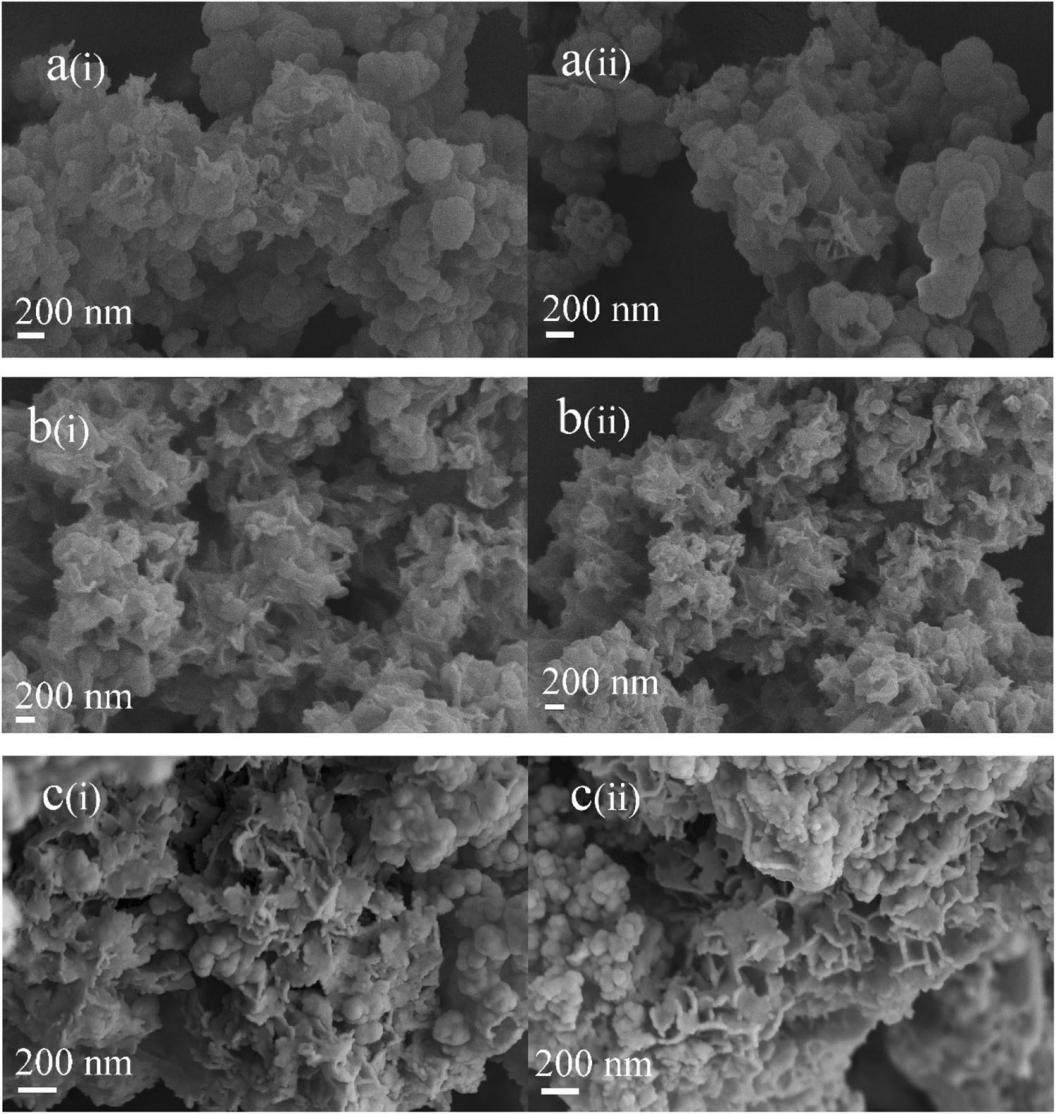
The SEM images of the BGNPs prepared by either (i) the RFNP or (ii) the tradition mixing after in vitro biodegradation for (**a**) 1, (**b**) 3, and (**c**) 7 days

With the optimized Ca/Si of 0.71 by the composition comparison in Fig. 5, the BGNP powder prepared by each method are immersed in an SBF solution for 1, 3, and 7 days to observe the deposition of carbonate hydroxyapatite (HCA) on BGNP surface. As shown in Fig. 6, the biodegradation of BGNPs happens in 1 day, and acicular HCA begins to appear on part of the particle surface, confirming the bioactivity of the BGNPs [3, 13, 33]. As the biodegradation time extends to 3 days, more HCA is observed. With the further biodegradation of the BGNPs in 7 day, leaf-like hydroxyapatite forms. The growth of hydroxyapatite on the BG surface indicates that the BGs prepared by both methods are bioactive.

**Fig. 7:**
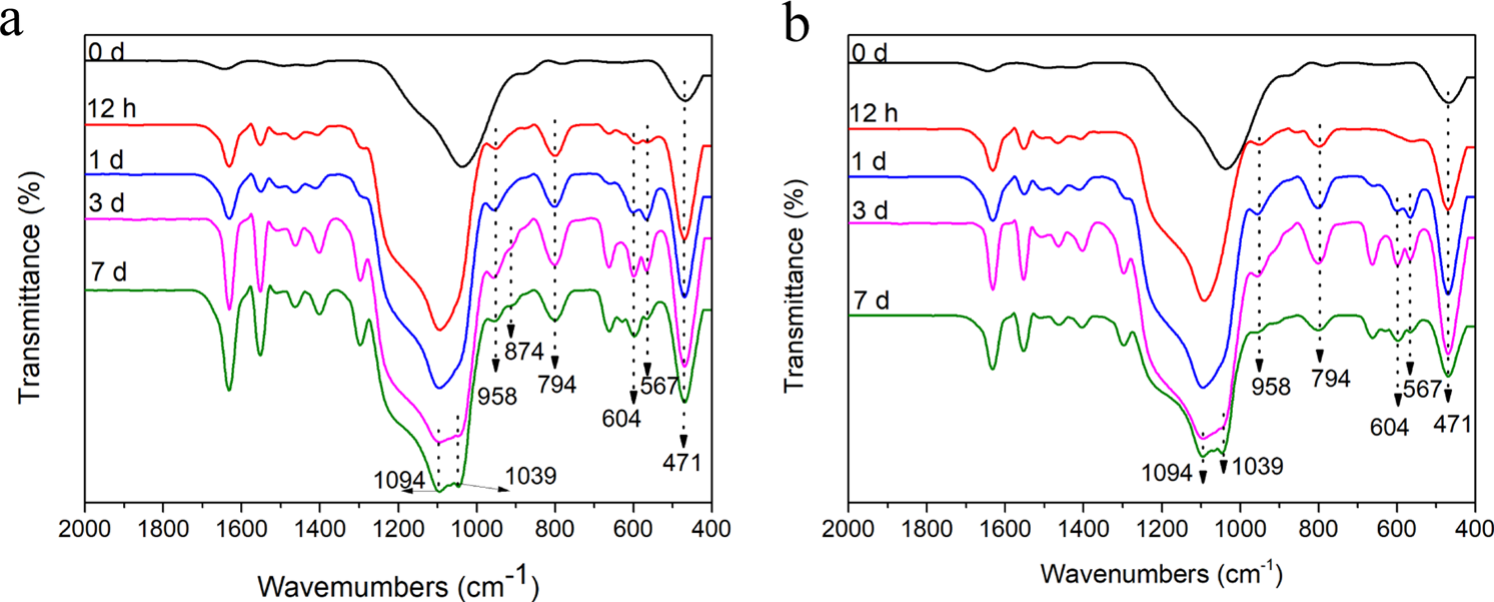
The infrared spectra of the BGNPs prepared by either (**a**) the RFNP or (**b**) the tradition mixing after a series of in vitro biodegradation time

After being immersed in an SBF solution for 12 h, a portion of the BGNP powder prepared by each method was taken out from the SBF solution for an FTIR measurement. A stretching vibration peak of P–O appears near 958 cm^−1^ appears in the FTIR spectrum for each specimen (Fig. 7). As the biodegradation time extends, the broad peak at 1000–1100 cm^−1^ gradually split into two peaks. One at 1094 cm^−1^ is a Si–O–Si vibration peak, and another at 1039 cm^−1^ is P=O stretching peak. After the samples eroded for 1 day, new double-shoulder absorption bands at 567 and 604 cm^−1^ appear. The double peaks are caused by the P–O bending vibration of PO_4_^3−^ in crystalline apatite [36]. However, after the BG specimens are immersed in SBF solution for 7 days, the strength of the double peaks gradually weakens, indicating that HCA is eroded after its formation. The reason could be that the BGNPs become smaller and smaller and the release rate and amount of calcium ions decrease significantly following the biodegradation and substitution process of SBF solution, which lead to the formation rate of HCA slower than its biodegradation rate [22].

**Fig. 8:**
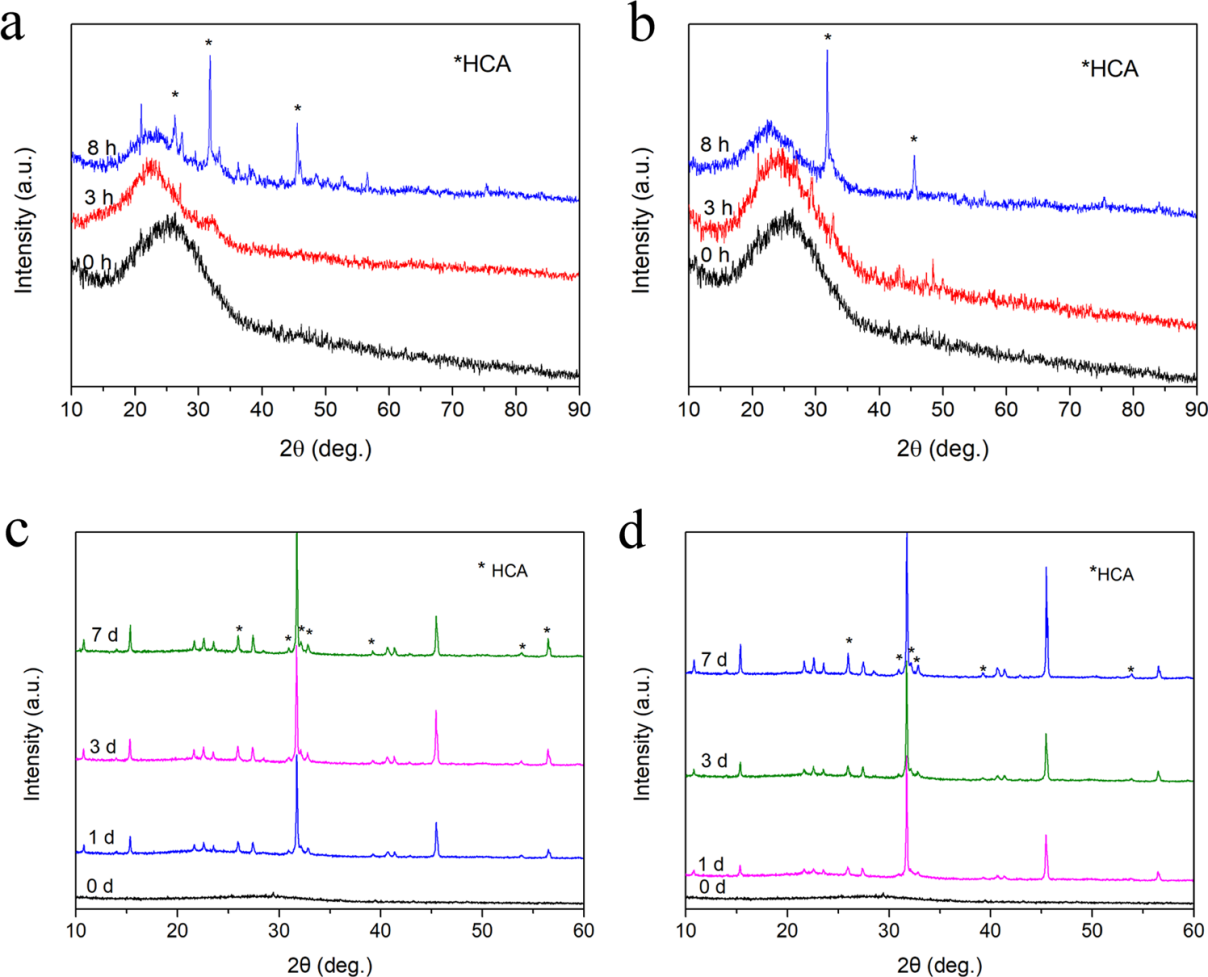
The XRD spectra of the BGNPs prepared by either (**a**, **c**) the RFNP or (**b**, **c**) the tradition mixing after a series of in vitro biodegradation time (**a**, **b**) 0, 3, and 8 h; (**c**, **d**) 0, 1, 3 and 7 days

As shown in Fig. 8, both the R-BGNPs and the T-BGNPs present a broad peak at 2*θ* of about 25° before the biodegradation, indicating that the BGNPs are in an amorphous state. As the biodegradation progresses, several characteristic sharp peaks appear at 2*θ* of 26, 32–35, 39, and 53^o^. Compared with the standard XRD spectrum of HCA (JCPDS 09–0432), these peaks correspond to the crystal faces of HCA, i.e., (002), (211, 112, 300, 202), (310), and (004), respectively [37, 38]. In 8 h, the R-BGNPs show more characteristic sharp peaks of HCA than the T-BGNPs (Fig. 8a, b), indicating that HCA forms on the R-BGNPs faster than on the T-BGNPs. This faster growth on the R-BGNPs suggests that the R-BGNPs have a greater bioactivity than the T-BGNPs. The reason could be that the R-BGNPs have a higher calcium content than the T-BGNPs, which also has been indicated by the composition comparision in Fig. 4. All of these results present that Ca^2+^ is evenly and sufficiently doped into the SiO_2_ matrix. The RFNP with fast mixing, chemical reaction and particle formation is an more effective way to wrap Ca^2+^ into SiO_2_ nanoparticles than the traditional mixing. After 1 day, the XRD peak intensity of the HCA forming on the two samples become stronger, and the XRD spectra tend to be consistent (Fig. 8c, d).

**Fig. 9:**
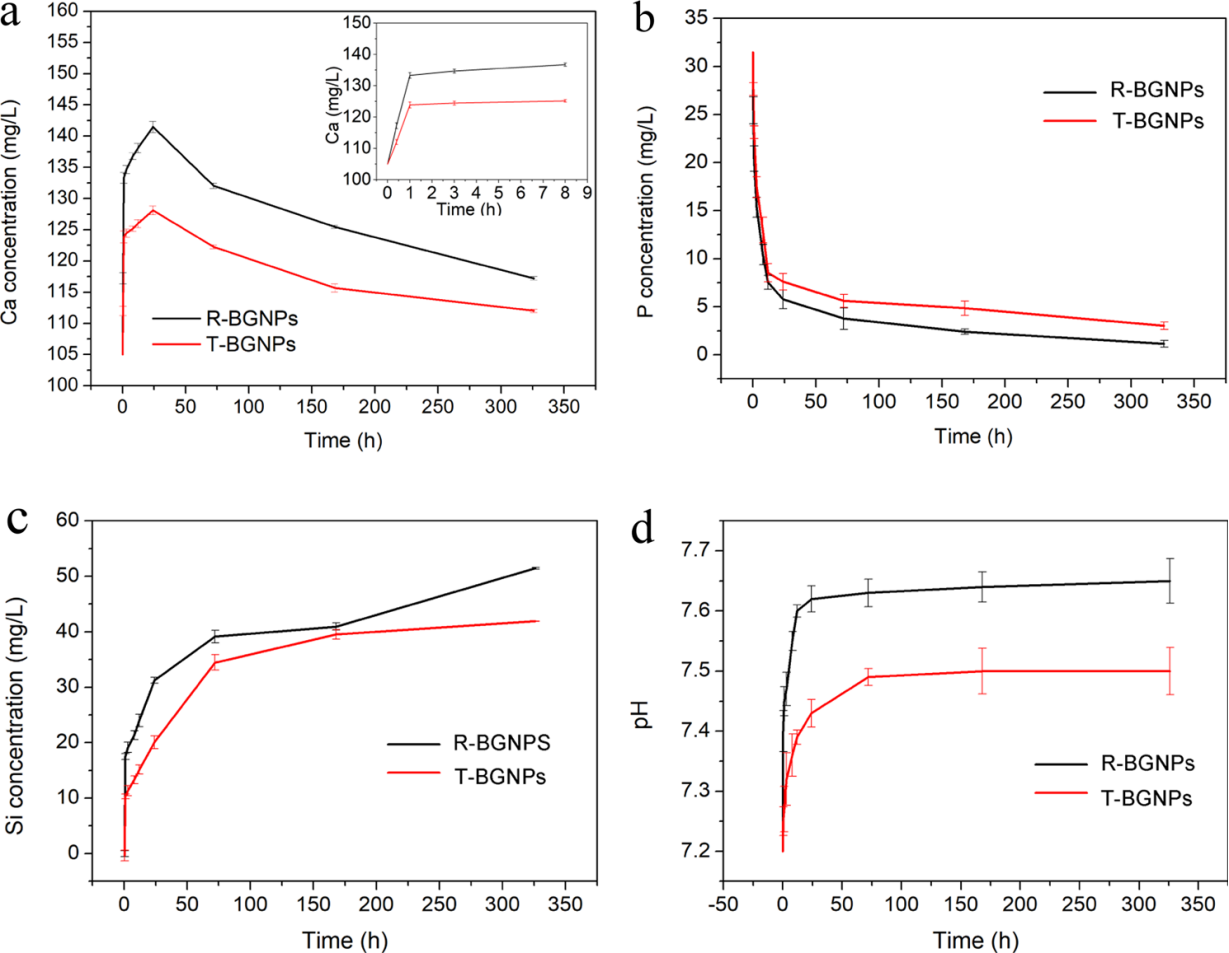
The ion release profiles of the BGNPs prepared by either the RFNP (R-BGNPs) or the traditional mixing (T-BGNPs): **a** Ca, **b** P, and **c** Si; **d** the pH change of the SBF aqueous medium

As shown in Fig. 9a, both the R-BGNPs and T-BGNPs begin to release Ca^2+^ rapidly within 20 min, and the Ca^2+^ concentration peaks at 24 h. Since hydroxyapatite begins to precipitate on the surface of the BGNPs as the Ca is released, as confirmed by our FTIR (Fig. 7) and XRD (Fig. 8) results, after 1 day the concentration of Ca^2+^ begins to decrease. The concentration of PO_4_^3+^ in the SBF solution begins to decrease as soon as the R-BGNPs and T-BGNPs are immersed into the SBF solution due to the formation of hydroxyapatite (Fig. 9b). The decrease of PO_4_^3+^ caused by the R-BGNPs is a little faster than that caused by T-BGNPs, suggesting that the bioactivity of the R-BGNPs is higher than the R-BGNPs. This result is consistent with that of XRD and FTIR characterization. After 7 days, the decrease of either Ca^2+^ or PO_4_^3+^ slows down, since the ion release and the precipitation of hydroxyapatite slower that at the peak. In Fig. 9c, d, either the release profile of Si or the pH change of the elution medium also suggests that the biodegradation of the R-BGNPs is faster than the one of the T-BGNPs.

## Conclusion

This work shows the superiority in terms of bioactivity of the BGNPs made by the RFNP with a high and even doping of calcium. The results show that the BGNPs made by the RFNP possessed a smaller size, narrower size distribution and more even composition than the ones by the traditional sol–gel method. The extremely fast and even mixing ensures the homogeneity of the chemical reaction and the succeeding formation of BGNPs. The sufficiently fast formation of the nanoparticles solidifies the blend state of calcium and SiO_2_, effectively represses the fast separation of calcium from the BGNPs, and brings out a composition of the BGNPs close to a feed formula. This work presents a superiority of the desired bioactivity of the BGNPs made by the RFNP with a high and even doping of calcium, and creates a way to effectively generate a high-quality material for bone repairs.
